# Enhancing Immunity and Gut Microbiota Balance with Black Soldier Fly Larvae (BSFL) Meal: A Sustainable Feed Ingredient That Maintains Growth Performance in Pekin Ducks

**DOI:** 10.3390/insects17060548

**Published:** 2026-05-25

**Authors:** Ling Long, Gaoqiang Liu, Guoji Gao, Gongtao Ding

**Affiliations:** 1 School of Bioengineering, Northwest Minzu University, Lanzhou 730030, China; 2 Key Laboratory of Biotechnology and Bioengineering of State Ethnic Affairs Commission, Biomedical Research Centre, Northwest Minzu University, Lanzhou 730030, China

**Keywords:** black soldier fly larvae, pekin ducks, protein feed, immune performance, gut microbiota

## Abstract

The effects of substituting soybean meal with black soldier fly larvae (BSFL) meal on the growth performance, immunological response, intestinal morphology, and gut microbiota of Pekin ducks were assessed. A total of 150 one-day-old Pekin ducks were randomly allocated to three isonitrogenous and isocaloric diets: a control diet (0% BSFL), and diets including 10% or 30% BSFL meal (T10 and T30, respectively). Over 42 days, growth metrics, organ indices, serum and jejunal immune parameters, intestinal morphology, and caecal microbiota composition were evaluated. Ducks fed BSFL-containing diets exhibited a significant increase in spleen index compared to the control group (0.06% ± 0.02% for T10 and T30 vs. 0.05% ± 0.01% for control, *p* < 0.05), while other organ indices and growth performance remained unaffected. Serum levels of immunoglobulins (IgG, IgM, sIgA), cytokines (IFN-γ, IL-2), and complements (C3, C4) were significantly elevated in both BSFL groups (*p* < 0.05). Jejunal immune indices, including IgG, IgM, IL-2, and C3, were also significantly higher in the T10 group (*p* < 0.05). Intestinal morphology showed a significantly increased villus-to-crypt ratio in the duodenum and jejunum (*p* < 0.05). Analysis of gut microbiota indicated that BSFL supplementation was associated with changes in caecal microbial composition, including a lower relative abundance of Proteobacteria. However, alpha diversity indices did not differ significantly among groups. These findings suggest that BSFL meal can replace soybean meal at inclusion levels up to 30% in Pekin duck diets without adversely affecting growth performance, while enhancing select immune parameters and modulating gut microbiota composition.

## Simple Summary

This study investigated whether black soldier fly larvae (BSFL) meal can be used as an alternative protein source to replace soybean meal in the diet of Pekin ducks. A total of 150 ducks were divided into three groups: one group received a standard diet, while the other two groups received diets in which 10% or 30% of the soybean meal was replaced with BSFL meal. Over 42 days, the researchers measured growth, immune function, intestinal health, and gut bacteria composition. The results showed that ducks fed BSFL meal had stronger immune responses, healthier intestinal structures, and a more beneficial gut microbial community, with no negative effects on growth. In particular, the 10% BSFL diet improved spleen function and gut immunity, while both 10% and 30% BSFL diets enhanced overall immune markers and modulated gut microbiota composition. These findings suggest that BSFL meal can effectively replace soybean meal in Pekin duck feed, supporting better health outcomes without compromising growth performance.

## 1. Introduction

The global demand for animal protein is increasing due to population growth and rising meat consumption, placing significant pressure on the livestock industry to enhance production efficiency [1]. Soybean meal (SBM), renowned for its high protein content and balanced amino acid profile, is the most widely used plant-based protein source in poultry diets [2]. However, limitations in soybean cultivation due to land scarcity and environmental concerns, and the availability of arable land for feed production, are diminishing, along with heavy reliance on imports in many countries, which have resulted in volatile supply and pricing, leading to challenges in supplying high-quality protein sources for livestock feeds [3].

To address these challenges, there is growing interest in exploring sustainable and unconventional protein sources to reduce reliance on traditional feeds such as SBM [4]. Insect meals have emerged as promising alternatives due to their efficient feed conversion, rapid reproduction, and high nutritional value [5,6,7]. Among these, black soldier fly larvae (BSFL; *Hermetia illucens*) have gained significant attention. BSFL can be cultivated on various organic waste substrates, transforming them into valuable biomass rich in protein and lipids, thereby promoting a circular economy and mitigating environmental impacts [8,9]. BSFL meal is distinguished by a high crude protein content (up to 50%), advantageous amino acid profiles, and bioactive substances, including antimicrobial peptides and medium-chain fatty acids (MCFAs), which may provide supplementary health benefits to animals [10,11]. Prior research has established the viability of integrating BSFL meal into aquaculture diets without negative impacts on growth performance or feed efficiency [2]. In poultry, BSFL meal has been assessed in broiler chickens, demonstrating the ability to partially substitute for SBM without detrimental effects on growth performance and potentially improving immune function and intestinal health [5,11].

However, research on the use of BSFL meal in duck diets, particularly in Pekin ducks, remains limited. Pekin ducks are economically important for meat production, and optimising their nutrition is crucial for sustainable production systems [12,13]. Given the distinct digestive physiology of ducks and their potential differences in nutrient utilisation compared with chickens, it is crucial to assess the impact of BSFL meal in duck diets. This study aimed to examine the impact of substituting SBM with BSFL meal at inclusion rates of 10% and 30% on the growth performance, immunological response, intestinal morphology, and gut microbiota of Pekin ducks. This study seeks to offer significant insights into the viability of incorporating BSFL meal into duck nutrition and to aid in the formulation of sustainable feeding strategies in poultry production.

We hypothesised that (1) BSFL meal can replace SBM up to 30% without adversely affecting growth performance (primary null hypothesis); (2) BSFL meal may enhance selected immune parameters and improve intestinal morphology (exploratory); (3) BSFL meal may be associated with changes in caecal microbiota composition, though causal inference is not claimed.

## 2. Materials and Methods

### 2.1. Experimental Design and Animal Management

The experiment was conducted at the duck facility of Lanzhou Guoruiyuan Agriculture Tech Co., Ltd., Lanzhou, China. A total of 150 one-day-old male Pekin ducks were obtained from a commercial hatchery. Ducks were randomly allocated to three dietary treatment groups with five replicates per group and ten ducks per replicate (*n* = 50 per group). For statistical analysis of growth performance variables, the pen (replicate) served as the experimental unit (*n* = 5 per treatment). For organ indices, immune parameters, intestinal morphology, and gut microbiota analysis, individual ducks were used as the experimental unit, with 10 ducks per treatment. Based on preliminary experiments, the experimental design included one control group (CK) and two treatment groups (T10 and T30). In the treatment groups, BSFL meal was added at 10% and 30% of the diet, respectively, partially replacing SBM and distillers’ grains to maintain isonitrogenicity. The full-fat dried BSFL (*H. illucens*) meal used in this study was purchased from Lanzhou Guorui Environmental Biotechnology Co., Ltd. (Lanzhou, China). The larvae were reared on a substrate composed of fruit and vegetable waste (60%), brewery spent grains (30%), and wheat bran (10%) for 14 days post-hatching. According to the manufacturer’s analysis, the composition was as follows: dry matter 83.00%, crude protein 47.70%, crude fat 34.80%, crude ash 14.20%, methionine 1.33%, lysine 4.62%, calcium 2.80%, and phosphorus 0.32%. All diets were formulated to be isonitrogenous and isocaloric, meeting or exceeding the nutritional requirements for Pekin ducks as per the NRC (1994) [14] and Chinese meat duck feeding standards (NY/T 2122-2012) [15]. Each duck was marked with a leg band for identification, and the experimental design is shown in Table 1. The feeding trial consisted of three stages: the brooding period (weeks 1–2), the growing period (weeks 3–5), and the fattening period (week 6). Ducks were housed in pens measuring 1.2 m × 1.2 m with a stocking density of approximately 7 ducks/m^2^. Each pen was equipped with a feeder and a drinker, allowing ad libitum access to feed and water. The duck house was environmentally controlled with automated ventilation, maintaining temperature and humidity appropriate for each growth stage. An 18 h light/6 h dark schedule was implemented throughout the study. All ducks were vaccinated according to standard veterinary protocols at the hatchery.

### 2.2. Organ Index and Growth Factor Detection in Breast and Leg Muscles

The selection of organ index and growth factors was based on prior studies showing that the source, proportion, and quality of protein in feed affect animal organ growth, immune function, and metabolism [5,16]. Insect protein substitution for conventional protein feeds (such as SBM) may influence organ growth and metabolism. Organ indices (e.g., heart, liver, spleen, lung, kidney) and growth factors insulin-like growth factor-I (IGF-I) and growth hormone receptor (GHR) were selected as detection indices due to their widespread use in assessing animal growth performance and health status. Growth factors, particularly IGF-I and GHR, are closely associated with muscle hypertrophy and metabolic processes, illustrating how nutritional intake influences growth efficacy. The heart, liver, spleen, lungs, and kidneys were removed and weighed on a precision electronic scale. Organ indices were calculated using the formula:

$$Organ\;Index=\left(\frac{Organ\;Weight}{Live\;Body\;Weight}\right)\times 100\%$$

The levels of IGF-I and GHR in breast and leg muscles were quantified using enzyme-linked immunosorbent assay (ELISA) with commercial kits from Shanghai Enzyme-linked Biotechnology Co., Ltd., adhering to the manufacturer’s instructions.

### 2.3. Serum and Jejunal Immune Parameters

Blood samples were obtained from the jugular vein into sterile tubes and centrifuged at 3000 rpm for 15 min to isolate serum, which was subsequently kept at −20 °C until analysis. The jejunum was dissected; mucosal samples were obtained by gentle scraping, homogenised, and then stored at −80 °C. Serum and jejunal samples were analysed for levels of immunoglobulins (IgA, IgG, IgM), cytokines (IFN-γ, IL-2), secretory immunoglobulin A (sIgA), and complements (C3, C4) utilising commercial ELISA kits (Shanghai Enzyme-linked Biotechnology Co., Ltd., Shanghai, China), in accordance with the manufacturer’s instructions.

### 2.4. Intestinal Sampling and Related Index Detection

At the end of the feeding trial, two ducks were randomly chosen from each replicate within each group. Segments (about 2 cm) of the duodenum, jejunum, and ileum were removed, rinsed with saline, and preserved in 4% paraformaldehyde. Tissues were dehydrated, embedded in paraffin, sectioned to 5 μm, and stained with haematoxylin and eosin (H&E). The tissues were visualised at 40× magnification utilising an Eclipse Ci-L microscope. The heights of five complete villi and the depths of five crypts were quantified using Image-Pro Plus 6.0 software and recorded in millimetres. The ratio of villus height to crypt depth was calculated to assess intestinal health.

### 2.5. Gut Microbiota Analysis

The experiment employed random group assignment, and individual differences were accounted for during data processing to mitigate potential errors. From each of the T10, T30, and control groups, 12 ducks were selected. Caecal contents were aseptically extracted, promptly frozen in liquid nitrogen and stored at −80 °C. Microbial DNA was isolated using a commercial DNA extraction kit from Sangon Biotech (Shanghai) Co., Ltd. (Shanghai, China). The V3–V4 segment of the 16S rRNA gene was amplified using universal primers 341F and 806R, with sample-specific barcodes. PCR products were purified, quantified, and sequenced using an Illumina MiSeq platform (Illumina, San Diego, CA, USA). A sequencing library was created, and 16S rDNA amplification sequencing analysis was conducted, with sequencing executed by Shanghai Meiji Biomedical Technology Co., Ltd., Shanghai, China. The Meiji Biomedical Cloud platform was utilised for data analysis. Sequences were grouped into operational taxonomic units (OTUs) at 97% similarity, and species identification was performed using the Greengenes database (version 13.8). Alpha and beta diversity were assessed to elucidate variations in microbiota composition among populations. The Chao method was employed to compute diversity indices at the OTU level. Principal coordinate analysis (PCoA) was conducted to examine variations in community structure among groups (i.e., beta diversity). The Kruskal–Wallis H test was employed to examine the differences in species among treatment groups.

### 2.6. Bioinformatics and Statistical Analysis

Sequencing data were analysed with QIIME software (Version 1.9.1). OTUs were grouped at 97% sequence similarity. Alpha diversity indices (Chao1, Shannon, Simpson) and beta diversity were computed. Taxonomic classification was conducted using the Greengenes database (Version 13.8). For differential abundance analysis of microbial taxa at phylum and genus levels, the Kruskal–Wallis H test was used, followed by pairwise comparisons with Benjamini–Hochberg false discovery rate (FDR) correction; adjusted *p*-values (*q*-values) < 0.05 were considered statistically significant. Beta diversity was further assessed using PCoA based on Bray–Curtis distances, and permutational multivariate analysis of variance was used to test for significant differences in microbial community structure among treatment groups. Statistical analyses for non-microbial data were performed using SPSS 21.0 (IBM Corp., Armonk, NY, USA). Data were assessed for normality and homogeneity of variance. One-way ANOVA was employed for normally distributed data, followed by Duncan’s multiple range test for post hoc analyses.

### 2.7. Ethical Considerations

All experimental procedures received approval from the Institutional Animal Care and Use Committee (IACUC) of Northwest Minzu University (Approval Number: xbmu-sm-202357) and were executed in compliance with national and international standards for the care and use of laboratory animals.

## 3. Results

### 3.1. Effect of BSFL Meal Substitution on Organ Indices in Pekin Ducks

The organ indices of Pekin ducks after 42 days of feeding are presented in Table 2. Ducks fed diets containing 10% and 30% BSFL meal exhibited a significant increase in spleen index compared to the control group (0.05% ± 0.01% and 0.06% ± 0.02% vs. 0.06% ± 0.02%, respectively; *p* < 0.05). No significant differences were observed among the groups for heart, liver, lungs, and kidney indices (*p* > 0.05), indicating that BSFL meal supplementation did not affect the relative weights of these organs. Notably, the increase in the spleen index, as an immune organ, may suggest enhanced immune function.

### 3.2. Effect of BSFL Meal on Muscle Growth Factors

The concentrations of IGF-I and GHR in the pectoral and leg muscles were assessed to determine the impact of BSFL meal supplementation on muscle growth in Table 3. Specifically, the IGF-I concentrations in the control group and the 10% BSFL group were 150.5 ± 4.8 and 154.3 ± 5.5 ng/mL, respectively, and in the 30% BSFL group, they were 152.0 ± 5.3 ng/mL. No significant differences were observed among the control, 10% BSFL, and 30% BSFL groups for IGF-I and GHR levels in both muscle types (*p* > 0.05). These findings suggest that replacing SBM with BSFL meal at inclusion levels of up to 30% does not adversely affect the expression of muscle growth factors in Pekin ducks. The lack of significant changes may be attributed to the diets being isonitrogenous and isocaloric, ensuring that the overall protein content and amino acid profiles remained consistent across treatments.

### 3.3. Effect of BSFL Meal on Serum and Jejunal Immune Parameters

**Figure 1:**
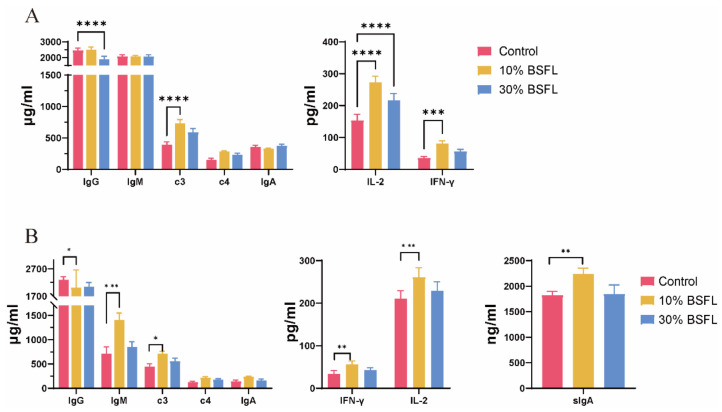
(**A**) Effect of BSFL meal on serum immune parameters in Pekin ducks; (**B**) effect of BSFL meal on jejunal immune parameters in Pekin ducks. Data are presented as mean ± SEM. * *p* < 0.05, ** *p* < 0.01, *** *p* < 0.001, **** *p* < 0.0001 compared to the control group.

Figure 1A illustrates the influence of varying BSFL additions on the serum immunological indices of Pekin ducks. Levels of IFN-γ, IL-2, C3, and C4 were markedly elevated (*p* < 0.05) in the 10% BSFL group relative to the control group, although IgA levels exhibited no significant variation (*p* > 0.05). In the 30% BSFL group, IL-2 and C3 levels were considerably increased (*p* < 0.05) relative to the control group, but IgA levels showed no significant differences (*p* > 0.05).

Figure 1B illustrates the influence of BSFL on jejunal immunological parameters. In the 10% BSFL group, concentrations of IgM, IL-2, and C3 were considerably elevated (*p* < 0.05) compared to the control group. In the 30% BSFL group, IL-2 and C3 levels were markedly elevated (*p* < 0.05), while the other indices exhibited no significant alterations. Further analysis suggests that the enhanced immune response observed in both the serum and the jejunum of the 10% BSFL group may be attributed to the rich functional components of BSFL, such as antimicrobial peptides and chitin, which can stimulate the immune system. While the higher BSFL level (30%) also led to significant improvements in some immune indices, the higher fibre and fat content may have inhibited further enhancement of certain immune responses.

### 3.4. Effect of BSFL Meal on Intestinal Morphology

**Figure 2:**
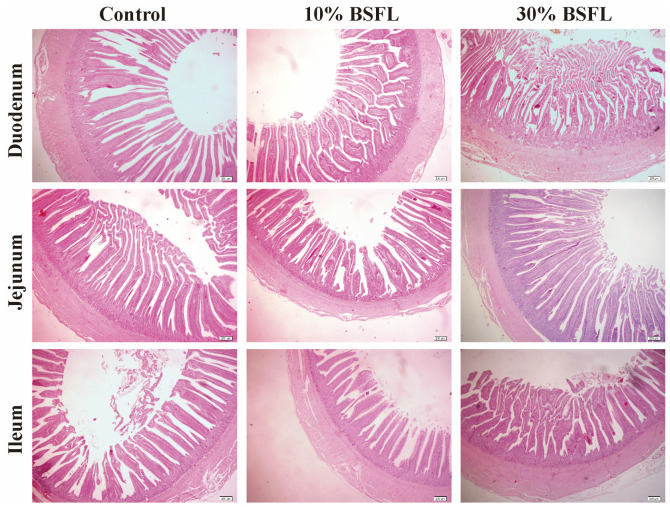
Histological sections of the duodenum, jejunum, and ileum in Pekin ducks. Representative H&E-stained sections of the duodenum from Control, 10% BSFL, and 30% BSFL at 200 μm.

Figure 2 and Table 4 illustrate that the incorporation of BSFL influenced the intestinal morphology of Pekin ducks. BSFL supplementation significantly increased the villus-to-crypt ratio in all three intestinal segments compared to the control group. In the duodenum, both T10 and T30 groups exhibited higher villus-to-crypt ratios than the control (6.54 ± 0.25 and 6.43 ± 0.24 vs. 5.40 ± 0.20, *p* = 0.026). Similarly, in the jejunum, T10 and T30 showed elevated ratios (6.38 ± 0.23 and 6.29 ± 0.23 vs. 6.00 ± 0.22, *p* = 0.038). In the ileum, the T10 group had a significantly higher ratio than the control group (6.09 ± 0.22 vs. 5.55 ± 0.21, *p* = 0.046), while the T30 group was intermediate and not significantly different from either group. Villus height and crypt depth showed numerical changes but did not reach statistical significance in any segment (*p* > 0.05). These results indicate that BSFL meal supplementation improves intestinal morphology primarily by increasing the villus-to-crypt ratio, which is associated with increased nutrient-absorption capacity.

### 3.5. Effect of Different Proportions of BSFL in the Diet on the Gut Microbiota of Pekin Ducks

#### 3.5.1. Analysis of Pan and Core Species Dilution Curves

**Figure 3:**
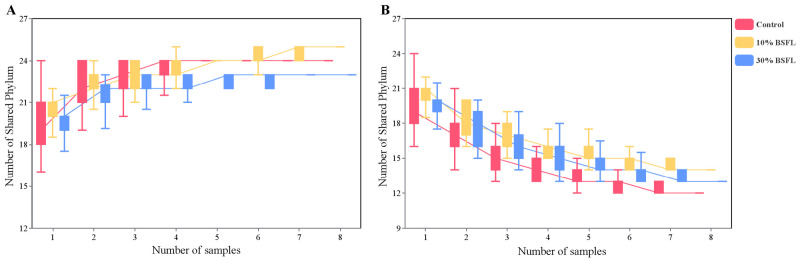
(**A**) The effect of different proportions of BSFL in the diet as a replacement for SBM on the Pan species curve in the caecum of Pekin ducks; (**B**) the effect of different proportions of BSFL in the diet as a replacement for SBM on the Core species curve in the caecum of Pekin ducks.

Based on all OTU data, dilution curves were plotted to assess the Pan species (all species) and Core species (core species). Pan OTUs denote the collective union of OTUs from all samples, whereas Core OTUs signify the OTUs that are common to all samples. Pan/Core species analysis can be conducted at multiple taxonomic levels and, with a substantial sample size, can assess sample adequacy while evaluating overall species richness and the number of core species present in the environment. The flatness of the Pan/Core species dilution curves indicates that as sequencing depth increases, the number of genes tends to stabilise. As shown in Figure 3A,B, the sequencing results indicate that the Pan and Core species dilution curves became flatter as sequencing depth increased, indicating that the sample size was sufficient to capture the main diversity of the gut microbiota and that the results were highly reliable. Furthermore, in the Pan species analysis, an increase in BSFL content in the diet (10% and 30% groups) was associated with a marginal increase in the overall species count relative to the control group, although not statistically significant. This suggests that BSFL supplementation may contribute to overall microbiota richness. However, the number of core species remained relatively stable across groups, indicating that the main bacterial communities in the gut were stable among treatments.

#### 3.5.2. Venn Diagram Analysis Based on OTU Counts

**Figure 4:**
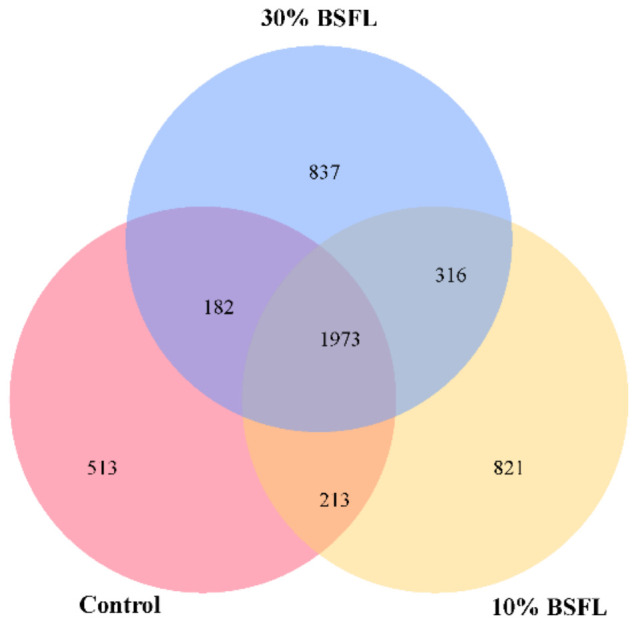
Venn diagram showing the number of OTUs in the caecum of Pekin ducks with different proportions of BSFL in the diet. Note: Different colours represent different groups. The petals show the number of unique species for each group, while the centre shows the number of species shared by all groups. Different colours in the above figure represent different subgroups, and the figure presents a petal diagram with the number of species specific to the corresponding subgroup in the petals and the number of species common to all subgroups in the centre.

A Venn diagram based on OTU data was drawn to show the distribution of shared and unique OTUs among the sample groups. As shown in Figure 4, the three treatment groups shared 1973 OTUs, with 513, 821, and 837 unique OTUs in each group, accounting for 5.6%, 4.1%, and 4.0%, respectively. This result indicates that although a substantial portion of the microbiota is shared between the groups, each group also has distinct microbial communities, reflecting the varying impact of BSFL supplementation on gut microbiota diversity. The number of unique OTUs did not differ significantly between the 10% and 30% BSFL groups, suggesting that the microbial population structure had stabilised between these two treatment groups. However, the unique microbial communities in each group may have specific metabolic functions, such as fibre degradation or amino acid metabolism, which could differentially impact the digestive efficiency of the ducks.

#### 3.5.3. Effects of BSFL on Intestinal Microbial Diversity in Meat Ducks

##### Alpha Diversity Analysis

**Figure 5:**
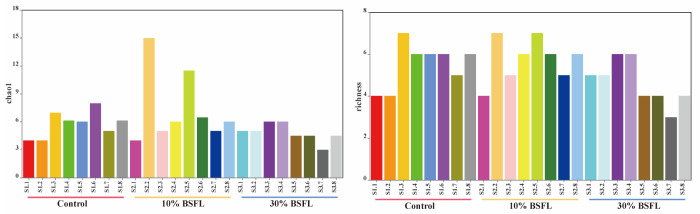
Alpha diversity indices of caecal microbiota in Pekin ducks. Note: The *x*-axis represents sample names, and the vertical coordinate is the alpha diversity index.

The alpha diversity indices reflect the diversity and richness of microbial communities, and commonly used metrics include Chao1, Shannon, Ace, Simpson, Coverage, and others. Alpha diversity indices (Chao1, Shannon, Simpson) showed no significant differences among the control, T10, and T30 groups (*p* > 0.05 for all indices; Figure 5). This indicates that BSFL supplementation at these inclusion levels did not alter the overall richness or evenness of the caecal microbial community.

##### Beta Diversity Analysis

**Figure 6:**
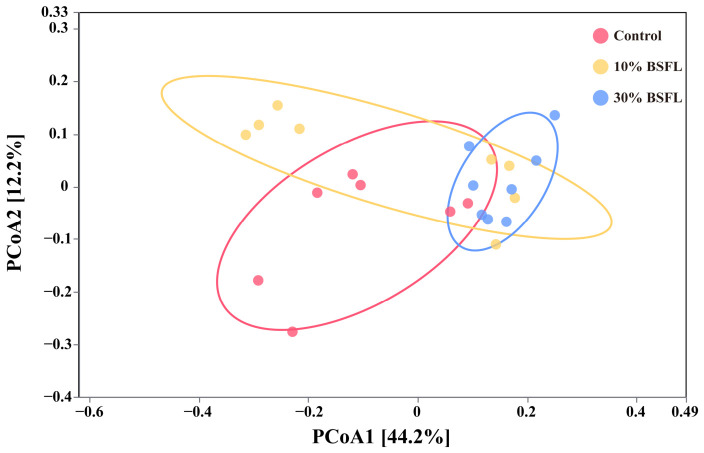
PCoA of caecal microbiota in Pekin ducks.

PCoA was employed to assess the similarity of species composition among groupings. Figure 6 shows that clustering of samples from the 10% and 30% BSFL groups was notably high, suggesting similar microbial community composition between these two groups. PERMANOVA on Bray–Curtis distances revealed no significant differences in overall microbial community structure among the three groups (R^2^ = 0.052, *p* = 0.183), indicating that the inclusion of BSFL did not substantially modify the overall microbiota structure at the genus level. The PCoA indicated structural similarities in the microbiota at the genus level among the groups; however, subsequent analysis showed that several significant genera exhibited varying degrees of increase or decrease across the treatment groups. The relative abundance of *Lactobacillus* and *Bacteroides* increased numerically in the 10% and 30% BSFL groups, but after FDR correction, these differences were not statistically significant (*q* > 0.05), whereas the abundance of certain potentially harmful bacteria diminished. This suggests that incorporating BSFL could positively influence the ecological equilibrium of the gut microbiota, thereby improving intestinal health and resilience in meat ducks.

#### 3.5.4. Effects of BSFL on Gut Microbiota

**Figure 7:**
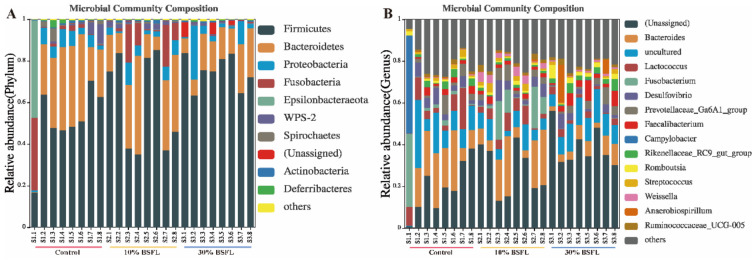
(**A**) Relative abundance of caecal microbiota at the phylum level; (**B**) Relative abundance of caecal microbiota at the genus level (top 10 species). The *x*-axis represents sample names, and the *y*-axis represents the proportion of species present in each sample. The differently coloured bars represent different species, and the bar lengths indicate the proportion of each species.

The outcomes of variations in relative abundance at the phylum and genus levels within the caecal microbiota of Pekin ducks subjected to various treatments are presented below. Figure 7A shows that, at the phylum level, the relative abundance in the caecal microbiota of Pekin ducks was dominated by Firmicutes, Bacteroidota, and Proteobacteria. The investigation focused on the 20 most prevalent species. The findings indicated that Firmicutes and Bacteroidota were the predominant phyla across all treatment groups. The prevalence of Firmicutes was greatest in the 30% BSFL group, suggesting that elevated BSFL supplementation may exert a more pronounced positive influence on beneficial bacteria. The prevalence of Proteobacteria, a phylum that includes both commensal and opportunistic pathogens, was lower in the 10% and 30% BSFL groups than in the control group, suggesting that BSFL supplementation may impede the proliferation of pathogenic bacteria. The heightened prevalence of Firmicutes may correlate with their role in fibre degradation and improved energy-use efficiency, whilst the diminished presence of Proteobacteria may signify improvements in the intestinal milieu.

Figure 7B shows the relative abundance of caecal microbiota at the genus level for each treatment group. The top 10 most abundant genera were selected for analysis. The results showed that the dominant genus in each group was *Bacteroides*. Although there was a numerical decrease in *Bacteroides* abundance in the 30% BSFL group compared to the control and 10% BSFL groups, this difference did not reach statistical significance after FDR correction (*q* > 0.05). This numerical trend may reflect a regulatory effect of BSFL on the gut microbiota, particularly a reduction in *Bacteroides*, which may be associated with alterations in nutrient structure or competitive advantages in the gut due to high levels of BSFL. It is worth noting that the reduction in *Bacteroides* may affect lipid and polysaccharide metabolism, and further research is needed to explore its potential impact on the metabolism and health of Pekin ducks. Additionally, analysing trends in other genera can further elucidate the overall impact of BSFL supplementation on the structure of the gut microbiota.

#### 3.5.5. Analysis of Significant Differences in Microbial Species Between Groups

**Figure 8:**
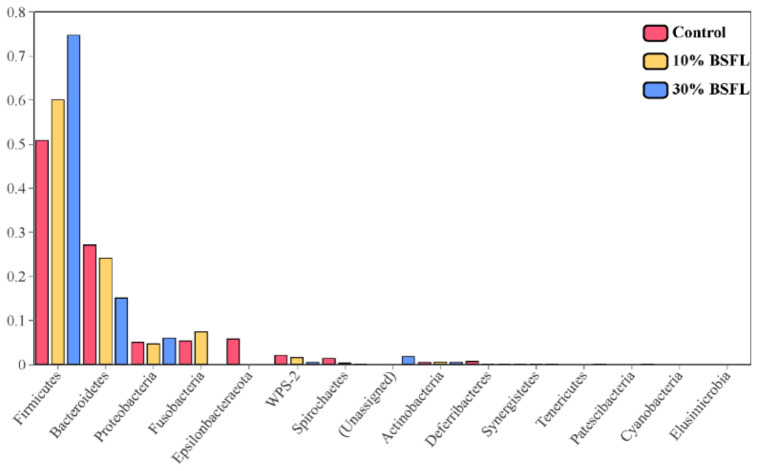
Kruskal–Wallis H test analysis of caecal microbiota at the genus level in Pekin ducks with different levels of BSFL substituted for SBM.

Significant differences in caecal microbiota at both the phylum and genus levels among the various treatment groups were assessed utilising the Kruskal–Wallis H test. Figure 8 shows that, while no significant variations were observed at the phylum level among the treatment groups, notable changes in the abundance of specific bacterial genera were evident. The abundance of potentially beneficial bacteria, such as *Lactobacillus*, increased in the BSFL-treated groups, whereas the abundance of some harmful bacteria decreased. However, after Benjamini–Hochberg FDR correction for multiple comparisons (15 genera tested), none of these genus-level differences were statistically significant (*q* > 0.05). While the overall introduction of BSFL produced negligible changes in caecal microbiota structure, modifications in certain bacterial groups could have substantial ramifications for gut health.

#### 3.5.6. Functional Prediction Analysis

##### COG Functional Classification and Abundance Analysis

**Figure 9:**
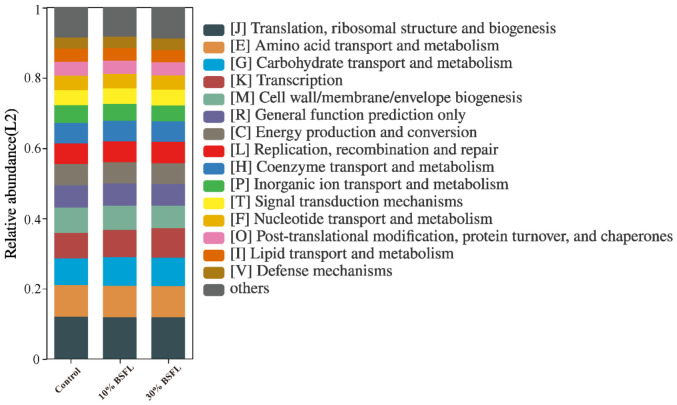
COG functional category abundance.

Following normalisation of OTU abundance via PICRUSt analysis, the findings indicated that the caecal microbiota across treatment groups exhibited higher predicted abundances of genes associated with ribosomal structure and biogenesis, amino acid transport and metabolism, and carbohydrate transport and metabolism (see Figure 9). The elevated prevalence of these functional pathways, particularly in amino acid and carbohydrate metabolism, suggests that incorporating BSFL may enhance nutrient absorption and metabolic efficiency in meat ducks. Relative to the control group, the prevalence of genes associated with amino acid metabolism was markedly elevated in the 30% BSFL group, suggesting that BSFL may augment the growth performance of ducks by enhancing food consumption efficiency. These data further support the potential use of BSFL as an alternative feed source.

##### KEGG Functional Classification and Metabolic Pathway Analysis

**Figure 10:**
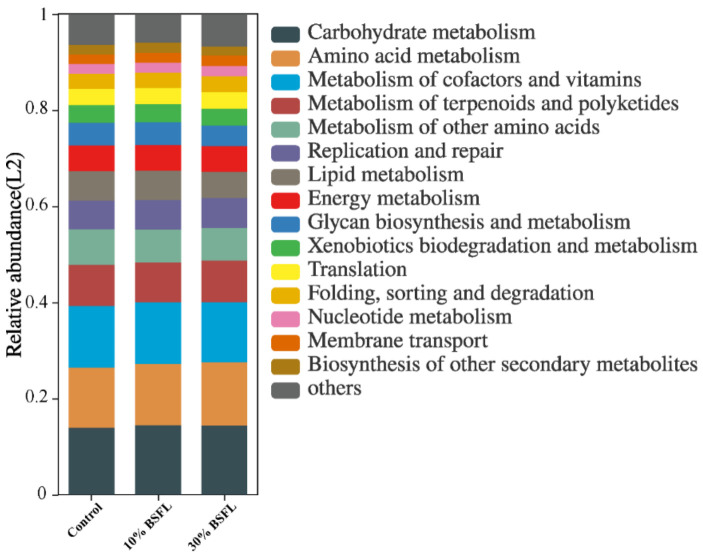
KEGG functional category abundance.

Based on KEGG functional predictions, the study further analysed the impact of BSFL addition on various metabolic pathways. As shown in Figure 10, in secondary metabolic pathways, carbohydrate metabolism, amino acid metabolism, and cofactor and vitamin metabolism were the main enriched pathways based on predicted functional gene abundance across the treatment groups. Notably, in the 30% BSFL group, the activity of these metabolic pathways was significantly higher than in the other groups, suggesting that a higher proportion of BSFL inclusion might improve the metabolic status of the meat ducks, thereby enhancing feed utilisation and growth rates. Furthermore, the regulatory effect of BSFL addition on specific metabolic pathways, such as amino acid and vitamin metabolism, may provide additional nutritional support for ducks, thereby promoting growth and production performance.

## 4. Discussion

The present study evaluated the effects of replacing SBM with BSFL meal at inclusion levels of 10% and 30% on the growth performance, immune response, intestinal morphology, and gut microbiota of Pekin ducks. The findings indicate that BSFL meal can serve as a viable alternative protein source in Pekin duck diets without adversely affecting growth performance while enhancing immune function and modulating gut health.

The significant increase in spleen index observed in ducks fed the 10% and 30% BSFL diets suggests an immunostimulatory effect of BSFL meal. The spleen plays a crucial role in the immune system as a site for lymphocyte proliferation and antibody production [17]. The increase in spleen weight may be attributed to bioactive components in BSFL meal, such as antimicrobial peptides and MCFAs, which can enhance immune responses [11]. Similar findings were reported by Gariglio et al. [18], where Muscovy ducks fed diets containing 3% to 9% BSFL meal showed no adverse effects on major organ weights, supporting the safety of BSFL meal inclusion in duck diets.

The lack of significant differences in IGF-I and GHR concentrations among the groups indicates that the BSFL meal did not affect muscle growth factors. This suggests that the isonitrogenous and isocaloric diets maintained consistent protein quality and amino acid profiles, resulting in comparable growth performance across all treatments [14]. These results align with those of Fiorilla et al. [19], who found that partial replacement of SBM with BSFL meal did not affect growth performance in broiler chickens.

The significant increases in serum immunoglobulins (IgG, IgM, sIgA), cytokines (IFN-γ, IL-2), and complements (C3, C4) in ducks fed BSFL diets indicate an enhanced immune response. These changes may result from the bioactive substances in BSFL, such as MCFAs and antimicrobial peptides, which have been reported in other studies to stimulate immune responses. which in turn increases the secretion of inflammatory factors like IL-2 and IFN-γ. These factors regulate the activation of T lymphocytes, B lymphocytes, macrophages, dendritic cells, and natural killer cells, thereby enhancing immunoglobulin concentrations. Immunoglobulins are critical components of the humoral immune system, protecting against pathogens [20]. Furthermore, the study found that adding 10% BSFL significantly increased the levels of IgG, IgM, IL-2, and C3 in the jejunum of Pekin ducks (*p* < 0.05). This may be related to the MCFAs in BSFL, which enhance immune function by activating receptors on the surface of macrophages [21]. The increase in immune factors such as IL-2, C3, and IFN-γ reflects the immune-boosting effects of BSFL, which further contribute to the elimination of harmful bacteria in the intestines [22]. The study by Liao et al. [21] showed that adding BSFL to the diet could increase IL-2 and C3 levels in koi fish. The research by Hartinger et al. [23] also indicated that adding 10% and 30% BSFL to trout diets did not significantly affect IL-2 expression in the jejunum, further demonstrating the beneficial regulatory role of BSFL in gut immunity. The elevation of these immune parameters suggests that the BSFL meal may enhance both systemic and mucosal immunity.

The bioactive compounds in BSFL meal, such as chitin and lauric acid (an MCFA), have been reported to possess immunomodulatory properties [24]. Chitin and its derivatives can stimulate cytokine production and activate immune cells, thereby enhancing host defence mechanisms [25]. Lauric acid has antimicrobial effects and can modulate immune responses by affecting cell signalling pathways [9]. IgG, as the main antibody in humoral immunity, has antibacterial and antitoxin functions [26], while IgM is the earliest antibody synthesised and secreted during poultry development, providing early humoral immune protection [20]. The significantly higher levels of IFN-γ and IL-2 in the BSFL-treated groups compared to the control indicate that BSFL can significantly stimulate the secretion of anti-inflammatory factors, promoting anti-inflammatory responses in the body. The increase in cytokines like IFN-γ and IL-2 further supports the immunostimulatory effect of BSFL meal, as these cytokines play key roles in activating immune cells and regulating immune responses [27].

The enhancement of intestinal morphology, evidenced by increased villus height and villus-to-crypt ratios in the duodenum and jejunum, suggests improved nutrient absorption capacity in ducks fed BSFL diets. Longer villi and higher villus-to-crypt ratios are associated with greater absorptive surface area and efficient nutrient utilisation. These morphological improvements may contribute to better overall gut health and digestion. The positive effects on intestinal structure may be linked to the bioactive components in BSFL meals. Chitin, found in the exoskeleton of BSFL, acts as a prebiotic fibre, promoting the proliferation of beneficial gut bacteria and enhancing gut barrier integrity. Moreover, antimicrobial peptides in BSFL may diminish harmful microorganisms in the gastrointestinal tract, hence reducing inflammation and promoting intestinal integrity [18].

This research employed 16S rDNA sequencing to assess the impact of varying BSFL dosages on caecal microbial diversity and bacterial community structure in Pekin ducks. The findings indicated that the number of OTUs in the 10% and 30% BSFL groups was higher than in the control group, but these differences were not statistically significant (*p* > 0.05). The MCFAs abundant in BSFL have strong antibacterial activity, especially lauric acid, which is speculated to be absorbed in the upper digestive tract and may help maintain the balance and health of the intestinal microbiota by inhibiting the proliferation of potentially pathogenic bacteria, regulating inflammatory responses, and promoting wound healing [28]. The gut microbiota plays a crucial role in the growth, nutrient absorption, and immune regulation of animals. A stable intestinal microecological environment not only supports nutrient synthesis and metabolism but also plays a key role in preventing various intestinal diseases [29].

The effect of BSFL on the microbial genus level in the caecum of Pekin ducks showed that the richness index increased numerically but did not reach statistical significance. In the Beta diversity analysis, the 10% and 30% BSFL groups were clearly separated from the control group, indicating differences in microbial community composition among the groups. Chitin in BSFL is thought to contribute to increased gut microbial richness and diversity by providing nutritional support for beneficial bacterial populations, thereby promoting their growth. The study by Guerreiro et al. [30] also found that feeding BSFL slightly increased the α-diversity of the gut microbiota in European sea bass and significantly impacted its microbial community composition. Higher diversity is often associated with the gut microbiota’s ability to resist potential pathogen invasion and helps regulate the host’s response under stress conditions. Similarly, Giulia Gaudioso et al. [31] showed that adding 8–45% BSFL had positive effects on the gut microbiota of trout. Therefore, the numerical increases in OTUs and the richness index observed in this experiment suggest that moderate BSFL supplementation may improve the structure of the gut microbiota, although statistical significance was not achieved.

We assessed caecal microbiota composition at the phylum and genus levels using species annotation results to determine the role of BSFL in maintaining homeostasis. At the phylum level, Firmicutes, Bacteroidota, and Proteobacteria accounted for over 80% of the microbial population, consistent with other research findings [24]. The abundance of Firmicutes in the 30% BSFL group was higher than in the other two groups, which may be related to the herbivorous characteristics of Pekin ducks. Firmicutes can break down cellulose into volatile fatty acids that can be utilised by the host, improving the digestion and utilisation of crude fibre [32]. Bacteroidota regulates intestinal pH by producing short-chain fatty acids, improving intestinal health, and inhibiting the proliferation of harmful gut bacteria. The study by Tiengtam et al. [33] found that BSFL supplementation reduced the abundance of pathogenic bacteria, including E. coli and Salmonella, in broilers. In this study, the abundance of Proteobacteria in the 10% and 30% BSFL groups was lower than in the control group, which indicates that the observed reduction in Proteobacteria abundance, a phylum that can contain opportunistic pathogens associated with dysbiosis, suggests that BSFL supplementation may contribute to a gut microbiota composition typically linked to lower disease risk [33,34].

*Bacteroides* was the predominant genus in the caecal microbiota. The abundance of *Bacteroides* decreased with increased BSFL intake, but the differences across groups were not statistically significant after FDR correction (*q* > 0.05). *Bacteroides* are crucial for energy production and lipid metabolism in the host, and their diminished abundance may influence lipid metabolism and the composition of the associated gut microbiota. Consequently, BSFL may indirectly influence gut health by modulating the prevalence of Bacteroidota and *Bacteroides*.

The composition and diversity of the gut microbiota are influenced by various factors, including diet, environment, community structure, age, and sex, and different taxa within the microbiota typically have distinct functional markers [28]. In this study, we observed that functional prediction analyses (COG and KEGG) revealed that the 30% BSFL group exhibited increased abundance of genes involved in carbohydrate metabolism, amino acid metabolism, and cofactor/vitamin metabolism (Figure 9 and Figure 10), providing complementary evidence that BSFL supplementation may enhance nutrient utilisation and metabolic efficiency. Increasing BSFL supplementation was associated with higher Firmicutes abundance and lower Proteobacteria abundance, possibly due to its ability to enhance the host’s immune response by regulating T cell activity, preventing intestinal inflammation, and maintaining microbial ecological balance. This observation aligns with the findings of Cutrignelli et al. [35], who reported that BSFL supplementation helps preserve gut microbial stability and reduces the likelihood of pathogen invasion. Research has established that the gut microbiota in animals is essential for host nutrition absorption, metabolism, and immunological control [36,37]. An in-depth examination of alterations in the functional capacities of the gut microbiota will enhance our comprehension of the adaptation processes employed by various hosts in response to these microorganisms. This study found no significant differences in the metabolic function of gut microbiota with increased BSFL intake, potentially due to the experiment’s duration or sample size. Subsequent research may employ longitudinal or larger-sample trials to further substantiate this finding.

The findings of this study demonstrate that BSFL meal can effectively replace SBM up to 30% in Pekin duck diets without adverse effects on growth performance. The immunostimulatory effects and improvements in gut health observed suggest additional benefits of the BSFL meal beyond its nutritional value. Incorporating BSFL meals into poultry diets aligns with sustainable feeding strategies, as BSFL can be produced on organic waste substrates, reducing environmental impact and contributing to a circular economy.

While the study provides valuable insights, certain limitations should be acknowledged. A limitation of our study design is that the inclusion of BSFL was accompanied by changes in other dietary components (DDG) to maintain isonitrogenicity. Therefore, the observed effects may reflect the combined dietary changes rather than BSFL alone. The duration of the experiment was 42 days, which may not capture the long-term effects of BSFL supplementation. Additionally, the study focused on specific inclusion levels (10% and 30%), and intermediate levels were not assessed. Future research should explore a wider range of inclusion levels, evaluate long-term effects, and consider economic analyses to determine the cost-effectiveness of BSFL meals in commercial duck production. Further investigations into the mechanisms underlying the immunostimulatory effects of BSFL meal are warranted. Studies examining the specific bioactive compounds responsible for the observed benefits, as well as their interactions with the gut microbiota, would provide a deeper understanding and facilitate the development of optimised diets.

## 5. Conclusions

In conclusion, the findings of this study demonstrate that incorporating BSFL meal into the diets of Pekin ducks at inclusion levels of 10% and 30% does not adversely affect growth performance, provided that diets are formulated to be isonitrogenous and isocaloric. Significant increases in serum and jejunal immune parameters (immunoglobulins, cytokines, complements) and improvements in the villus-to-crypt ratio in the duodenum and jejunum suggest that the BSFL meal may confer immunomodulatory and gut health benefits. Analysis of the caecal microbiota revealed changes in composition, including a lower relative abundance of Proteobacteria, although alpha diversity was not significantly altered. These results support the potential of BSFL meal as a partial replacement for SBM in Pekin duck diets. However, future studies are needed to confirm the causal relationships between BSFL supplementation, immune enhancement, and gut microbiota modulation, and to evaluate the economic and environmental sustainability of BSFL meal in commercial duck production.

## Figures and Tables

**Table 1:** Composition and calculated nutritional content of experimental diets (% as-fed basis).

Component	Treatment		
	Control	T10	T30
Dried BSFL/%	0	10	30
Corn/%	50	50	50
Distillers’ grains/%	12	10	6
SBM/%	27	19	3
Wheat bran/%	5	5	5
NaCl/%	1.37	1.37	1.37
Limestone powder/%	0.82	0.82	0.82
Premix/%	3.2	3.2	3.2
CaHPO_4_/%	0.61	0.61	0.61
Metabolizable energy (MJ/kg)	12.53	12.56	12.59
Crude protein (%)	17.66	17.55	17.33
Calcium (%)	1.55	1.55	1.55
Available phosphorus (%)	0.68	0.68	0.68
Methionine (%)	0.47	0.48	0.48
Lysine (%)	1.15	1.16	1.18

Note: The premix (3.2% of the diet) supplied per kilogram: Vitamin A 8000 IU, vitamin D3 1000 IU, vitamin E 20 IU, vitamin K3 0.5 mg, vitamin B1 2.0 mg, vitamin B2 8.00 mg, vitamin B6 3.50 mg, vitamin B12 0.01 mg, niacin 35.00 mg, pantothenic acid 10.00 mg, folic acid 0.55 mg, biotin 0.18 mg, iron 80 mg, copper 8 mg, manganese 80 mg, zinc 60 mg, iodine 0.35 mg, selenium 0.15 mg. Metabolic energy was calculated based on the raw material composition, and other indexes were measured values.

**Table 2:** Effect of BSFL meal on organ indices of Pekin ducks.

Organ	Control (%)	T10 (%)	T30 (%)	*p*-Value
Heart	0.46 ± 0.01	0.47 ± 0.02	0.48 ± 0.01	0.624
Liver	1.66 ± 0.05	1.72 ± 0.07	1.64 ± 0.05	0.581
Spleen	0.05 ± 0.01 ᵇ	0.06 ± 0.02 ᵃ	0.06 ± 0.02 ᵃ	0.042
Lungs	0.86 ± 0.02	0.88 ± 0.03	0.85 ± 0.02	0.713
Kidneys	0.59 ± 0.02	0.64 ± 0.04	0.61 ± 0.02	0.408

Values are presented as mean ± standard error (*n* = 10 per group). Within a row, means with different superscript letters (a, b) differ significantly (*p* < 0.05).

**Table 3:** Effect of BSFL meal on IGF-I and GHR concentrations in pectoral and leg muscles.

Parameter	Control	T10	T30	*p*-Value
Pectoral Muscle				
IGF-I (ng/mL)	150.5 ± 4.8	154.3 ± 5.5	152.0 ± 5.3	0.874
GHR (ng/mL)	12.8 ± 0.4	13.0 ± 0.5	12.9 ± 0.4	0.949
Leg Muscle				
IGF-I (ng/mL)	145.7 ± 4.8	146.9 ± 5.0	146.2 ± 4.9	0.985
GHR (ng/mL)	12.5 ± 0.3	12.6 ± 0.4	12.5 ± 0.4	0.976

**Table 4:** Effect of BSFL meal on intestinal morphology of Pekin ducks.

Parameter	Control	T10	T30	*p*-Value
Villus height				
Duodenum (mm)	0.81 ± 0.02	0.85 ± 0.03	0.90 ± 0.03	0.081
Jejunum (mm)	0.78 ± 0.02	0.83 ± 0.03	0.88 ± 0.03	0.166
Ileum (mm)	0.61 ± 0.02	0.67 ± 0.02	0.68 ± 0.02	0.089
Crypt depth				
Duodenum (mm)	0.15 ± 0.01	0.13 ± 0.01	0.14 ± 0.01	0.562
Jejunum (mm)	0.13 ± 0.01	0.13 ± 0.01	0.14 ± 0.01	0.259
Ileum (mm)	0.10 ± 0.01	0.11 ± 0.01	0.13 ± 0.01	0.093
Villus-to-crypt ratio				
Duodenum (mm)	5.40 ± 0.20 ^b^	6.54 ± 0.25 ᵃ	6.43 ± 0.24 ᵃ	0.026
Jejunum (mm)	6.00 ± 0.22 ᵇ	6.38 ± 0.23 ᵃ	6.29 ± 0.23 ᵃ	0.038
Ileum (mm)	5.55 ± 0.21 ᵇ	6.09 ± 0.22 ᵃ	5.67 ± 0.21 ᵇ	0.046

Values are presented as mean ± standard error (*n* = 10 per group). Within a row, means with different superscript letters differ significantly (*p* < 0.05).

## Data Availability

The data presented in this study are available on request from the corresponding author due to privacy/ethical restrictions.

## References

[1] Makokha M.P., Muliro P.S., Ngoda P.N., Ghemoh C.J., Xavier C., Tanga C.M. (2023). Nutritional Quality of Meat from Hen Fed Diet with Full-Fat Black Soldier Fly (*Hermetia illucens*) Larvae Meal as a Substitute to Fish Meal. J. Funct. Foods.

[2] Eide L.H., Rocha S.D.C., Morales-Lange B., Kuiper R.V., Dale O.B., Djordjevic B., Hooft J.M., Øverland M. (2024). Black Soldier Fly Larvae (*Hermetia illucens*) Meal Is a Viable Protein Source for Atlantic Salmon (*Salmo salar*) during a Large-Scale Controlled Field Trial under Commercial-like Conditions. Aquaculture.

[3] Foley J.A., Ramankutty N., Brauman K.A., Cassidy E.S., Gerber J.S., Johnston M., Mueller N.D., O’Connell C., Ray D.K., West P.C. (2011). Solutions for a Cultivated Planet. Nature.

[4] Onono J.O., Alarcon P., Karani M., Muinde P., Akoko J.M., Maud C., Fevre E.M., Häsler B., Rushton J. (2018). Identification of Production Challenges and Benefits Using Value Chain Mapping of Egg Food Systems in Nairobi, Kenya. Agric. Syst..

[5] Tahamtani F.M., Ivarsson E., Wiklicky V., Lalander C., Wall H., Rodenburg T.B., Tuyttens F.A.M., Hernandez C.E. (2021). Feeding Live Black Soldier Fly Larvae (*Hermetia illucens*) to Laying Hens: Effects on Feed Consumption, Hen Health, Hen Behavior, and Egg Quality. Poult. Sci..

[6] Lu S., Taethaisong N., Meethip W., Surakhunthod J., Sinpru B., Sroichak T., Archa P., Thongpea S., Paengkoum S., Purba R.A.P. (2022). Nutritional Composition of Black Soldier Fly Larvae (*Hermetia illucens* L.) and Its Potential Uses as Alternative Protein Sources in Animal Diets: A Review. Insects.

[7] Alagappan S., Rowland D., Barwell R., Mantilla S.M.O., Mikkelsen D., James P., Yarger O., Hoffman L.C. (2022). Legislative Landscape of Black Soldier Fly (*Hermetia illucens*) as Feed. J. Insects Food Feed.

[8] Leni G., Cirlini M., Jacobs J., Depraetere S., Gianotten N., Sforza S., Dall’Asta C. (2019). Impact of Naturally Contaminated Substrates on *Alphitobius diaperinus* and *Hermetia illucens*: Uptake and Excretion of Mycotoxins. Toxins.

[9] Liu G., Tiang M.F., Ma S., Wei Z., Liang X., Sajab M.S., Abdul P.M., Zhou X., Ma Z., Ding G. (2024). An Alternative Peptone Preparation Using *Hermetia illucens* (Black Soldier Fly) Hydrolysis: Process Optimization and Performance Evaluation. PeerJ.

[10] Yan C., Xiao J., Li Z., Liu H., Zhao X., Liu J., Chen S., Zhao X. (2021). Exogenous Fecal Microbial Transplantation Alters Fearfulness, Intestinal Morphology, and Gut Microbiota in Broilers. Front. Vet. Sci..

[11] Huang C., Hernandez C.E., Wall H., Tahamtani F.M., Ivarsson E., Sun L. (2024). Live Black Soldier Fly (*Hermetia illucens*) Larvae in Feed for Laying Hens: Effects on Hen Gut Microbiota and Behavior. Poult. Sci..

[12] Guo R., Zeng T., Wang D., Zhao A., Zhou S., Huang Z., Chang Y., Sun H., Gu T., Chen L. (2024). Comparative Analysis of the Hypothalamus Transcriptome of Laying Ducks with Different Residual Feeding Intake. Poult. Sci..

[13] Liu H., Zhu C., Wang Y., Wang Z., Zou K., Song W., Tao Z., Xu W., Zhang S., Wang Z. (2024). Effects of Residual Feed Intake on the Economic Traits of Fast-Growing Meat Ducks. Poult. Sci..

[14] National Research Council (NRC) (1994). Nutrient Requirements of Poultry.

[15] (2012). Nutrient Requirements of Meat-Type Duck.

[16] Colombino E., Gariglio M., Biasato I., Ferrocino I., Pozzo S., Fragola E., Battisti E., Zanet S., Ferroglio E., Capucchio M.T. (2024). Insect Live Larvae as a New Nutritional Model in Duck: Effects on Gut Health. Anim. Microbiome.

[17] Xu X., Zhao J., Dou Y., Wei Z. (2019). Study on Improving the Nutritional Value of the Oil of the Black Soldier Fly by Using the Residue of Schizoite Alga. Feed. Ind..

[18] Gariglio M., Dabbou S., Gai F., Trocino A., Xiccato G., Holodova M., Gresakova L., Nery J., Bellezza Oddon S., Biasato I. (2021). Black Soldier Fly Larva in Muscovy Duck Diets: Effects on Duck Growth, Carcass Property, and Meat Quality. Poult. Sci..

[19] Fiorilla E., Gariglio M., Gai F., Zambotto V., Bongiorno V., Cappone E.E., Biasato I., Bergagna S., Madrid J., Martinez-Miró S. (2024). Dehydrated and Live Black Soldier Fly Larvae as Environmental Enrichment in Indigenous Slow-Growing Chickens: Performance, Gut Health, and Chitinolytic Enzyme Activity. Animal.

[20] Abbas S.K., Abdullah A.S. (2019). Lymphocytosis and Increases in Complement Protein C3 Level in Women with Polycystic Ovarian Syndrome. Indian J. Public Health Res. Dev..

[21] Liao X.D., Ma G., Cai J., Fu Y., Yan X.Y., Wei X.B., Zhang R.J. (2015). Effects of *Clostridium butyricum* on Growth Performance, Antioxidation, and Immune Function of Broilers. Poult. Sci..

[22] Smith K.A. (1988). Interleukin-2: Inception, Impact, and Implications. Science.

[23] Hartinger K., Greinix J., Thaler N., Ebbing M.A., Yacoubi N., Schedle K., Gierus M. (2021). Effect of Graded Substitution of Soybean Meal by *Hermetia illucens* Larvae Meal on Animal Performance, Apparent Ileal Digestibility, Gut Histology and Microbial Metabolites of Broilers. Animals.

[24] Attia Y.A., Bovera F., Asiry K.A., Alqurashi S., Alrefaei M.S. (2023). Fish and Black Soldier Fly Meals as Partial Replacements for Soybean Meal Can Affect Sustainability of Productive Performance, Blood Constituents, Gut Microbiota, and Nutrient Excretion of Broiler Chickens. Animals.

[25] Zhang C., Li C., Shao Q., Chen W., Ma L., Xu W., Li Y., Huang S., Ma Y. (2021). Effects of Glycyrrhiza Polysaccharide in Diet on Growth Performance, Serum Antioxidant Capacity, and Biochemistry of Broilers. Poult. Sci..

[26] Ehrenstein M.R., Notley C.A. (2010). The Importance of Natural IgM: Scavenger, Protector and Regulator. Nat. Rev. Immunol..

[27] Zhu X., Tao L., Liu H., Yang G. (2023). Effects of Fermented Feed on Growth Performance, Immune Organ Indices, Serum Biochemical Parameters, Cecal Odorous Compound Production, and the Microbiota Community in Broilers. Poult. Sci..

[28] Sypniewski J., Kierończyk B., Benzertiha A., Mikołajczak Z., Pruszyńska-Oszmałek E., Kołodziejski P., Sassek M., Rawski M., Czekała W., Józefiak D. (2020). Replacement of Soybean Oil by *Hermetia illucens* Fat in Turkey Nutrition: Effect on Performance, Digestibility, Microbial Community, Immune and Physiological Status and Final Product Quality. Br. Poult. Sci..

[29] Huang B., Zhang S., Dong X., Chi S., Yang Q., Liu H., Tan B., Xie S. (2022). Effects of Fishmeal Replacement by Black Soldier Fly on Growth Performance, Digestive Enzyme Activity, Intestine Morphology, Intestinal Flora and Immune Response of Pearl Gentian Grouper (*Epinephelus fuscoguttatus* ♀ × *Epinephelus lanceolatus* ♂). Fish Shellfish Immunol..

[30] Guerreiro I., Serra C.R., Oliva-Teles A., Enes P. (2018). Short Communication: Gut Microbiota of European Sea Bass (*Dicentrarchus labrax*) Is Modulated by Short-Chain Fructooligosaccharides and Xylooligosaccharides. Aquac. Int..

[31] Gaudioso G., Marzorati G., Faccenda F., Weil T., Lunelli F., Cardinaletti G., Marino G., Olivotto I., Parisi G., Tibaldi E. (2021). Processed Animal Proteins from Insect and Poultry By-Products in a Fish Meal-Free Diet for Rainbow Trout: Impact on Intestinal Microbiota and Inflammatory Markers. Int. J. Mol. Sci..

[32] Guan Y., Yang H., Han S., Feng L., Wang T., Ge J. (2017). Comparison of the Gut Microbiota Composition between Wild and Captive Sika Deer (*Cervus nippon hortulorum*) from Feces by High-Throughput Sequencing. AMB. Express.

[33] Tiengtam N., Khempaka S., Paengkoum P., Boonanuntanasarn S. (2015). Effects of Inulin and Jerusalem Artichoke (*Helianthus tuberosus*) as Prebiotic Ingredients in the Diet of Juvenile Nile Tilapia (*Oreochromis niloticus*). Anim. Feed Sci. Technol..

[34] Shin N.-R., Whon T.W., Bae J.-W. (2015). Proteobacteria: Microbial Signature of Dysbiosis in Gut Microbiota. Trends Biotechnol..

[35] Cutrignelli M.I., Messina M., Tulli F., Randazzo B., Olivotto I., Gasco L., Lussignoli M., Bovera F. (2017). Evaluation of an Insect Meal of the Black Soldier Fly (*Hermetia illucens*) as Soybean Substitute: Intestinal Morphometry, Enzymatic and Microbial Activity in Laying Hens. Res. Vet. Sci..

[36] Moniello G., Ariano A., Panettieri V., Tulli F., Olivotto I., Messina M., Randazzo B., Severino L., Piccolo G., Musco N. (2019). Intestinal Morphometry, Enzymatic and Microbial Activity in Laying Hens Fed Different Levels of a *Hermetia illucens* Larvae Meal and Toxic Elements Content of the Insect Meal and Diets. Animals.

[37] Li L., lv X., Han X., Sun C., An K., Gao W., Xia Z. (2022). Effect of Dietary *Bacillus licheniformis* Supplementation on Growth Performance and Microbiota Diversity of Pekin Ducks. Front. Vet. Sci..

